# Epigenetic Regulation of Higher-Order Chromatin Structure (HOCS) and Its Implication in Human Diseases

**DOI:** 10.3390/cancers18030483

**Published:** 2026-01-31

**Authors:** Luisa Ladel, Bethsebie Sailo, Paromita Das, Ethan Samuel Lin, Wan Ying Tan, Ankit Chhoda, Haoyu Tang, Olivia Ang-Olson, Linda He, Nithyla John, Jeremy D. Kratz, Anup Sharma, Nita Ahuja

**Affiliations:** 1 Surgical Oncology Research Laboratories, Department of Surgery, Yale School of Medicine, New Haven, CT 06510, USA; luisa.ladel@yale.edu (L.L.); sailo@wisc.edu (B.S.); paromita.1991@gmail.com (P.D.); wtan@uchc.edu (W.Y.T.); haoyu.tang@yale.edu (H.T.); olivia.ang-olson@yale.edu (O.A.-O.); linda.he@yale.edu (L.H.); njohn1@bronxcare.org (N.J.); 2 Department of Internal Medicine, Norwalk Hospital, Norwalk, CT 06856, USA; 3 Center for Precision Medicine, University of Wisconsin-Madison, Madison, WI 53705, USA; eslin2@wisc.edu (E.S.L.); jdkratz@medicine.wisc.edu (J.D.K.); 4 Department of Surgery, University of Wisconsin-Madison, Madison, WI 53705, USA; 5 Department of Medicine, Division of Hematology, Oncology and Palliative Care, University of Wisconsin-Madison, Madison, WI 53705, USA; 6 William S. Middleton Memorial VA Administration Health System, Madison, WI 53705, USA; 7 Department of Hematology & Oncology, Neag Comprehensive Cancer Center, UConn Health, Farmington, CT 06030, USA; 8 Department of Gastroenterology and Hepatology, Mayo Clinic, Rochester, MN 55905, USA; chhoda.ankit@mayo.edu; 9 Department of Surgery, BronxCare Health System, Bronx, NY 10457, USA; 10 Department of Pathology, Yale School of Medicine, New Haven, CT 06510, USA; 11 Yale Cancer Center, Yale School of Medicine, New Haven, CT 06510, USA; 12 Carbone Cancer Center, University of Wisconsin-Madison, Madison, WI 53705, USA

**Keywords:** higher-order chromatin structure, architectural proteins, nucleosomes, cancer, CCCTC-binding factor, cohesin, topologically associating domains, polycomb repressive complex

## Abstract

Higher-order chromatin structures (HOCS) are fundamental to genome organization, gene regulation, and cellular homeostasis. This review examines the epigenetic mechanisms shaping HOCS, including DNA methylation, histone modifications, chromatin remodeling, and RNA-based regulatory processes. We also discuss the role of architectural proteins in maintaining chromatin topology while allowing dynamic changes to chromatin structure, thereby influencing gene expression. Growing evidence indicates that disruptions in HOCS contribute to a diverse array of human diseases, including cancer, aging-related disorders, and congenital abnormalities, primarily through aberrant gene regulation. We further discuss the concept of distinct genomic areas, in which specific chromatin regions orchestrate three-dimensional (3D) genome dynamics, positioning them as potential biomarkers and therapeutic targets. By emphasizing chromatin architecture on a global scale rather than at the level of individual genes, this review underscores its emerging relevance to precision medicine. Finally, we synthesize current technical advances, outline future directions for leveraging chromatin topology in disease diagnosis and treatment, and highlight key biological insights to reshape our understanding of genome function.

## Simple Summary

This review explores how DNA folds into 3D higher-order chromatin structures that regulate gene activity. It highlights how epigenetic mechanisms and architectural proteins work together to shape the dynamic chromatin folding while allowing structural flexibility based on cellular needs. Disruption of these folding patterns leads to aberrant gene regulation, contributing to cancer, aging-related disorders, and certain congenital conditions. We emphasize specific genomic regions and epigenetic modulators that act as regulatory hubs for 3D organization, which could serve as promising biomarkers or therapeutic targets for cancer. Overall, it underscores the importance of a deeper understanding of DNA’s large-scale 3D architecture for advancing precision medicine and developing novel diagnostic approaches for cancer and other human diseases.

## 1. Introduction

**Figure 1:**
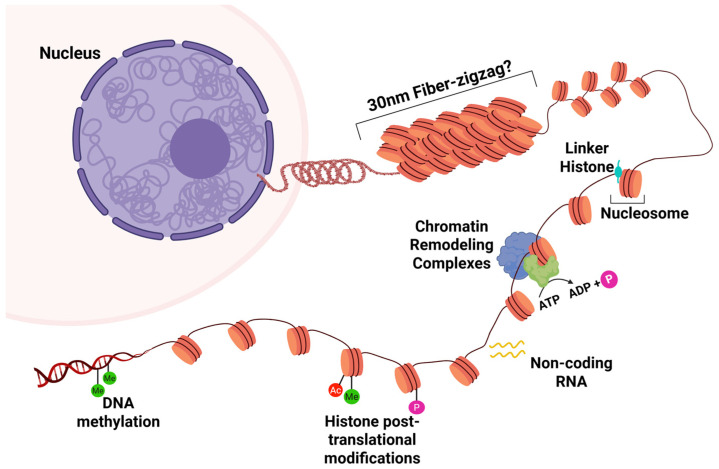
Overview of multiscale organization and epigenetic regulation of chromatin architecture. The chromatin fiber is spatially organized into an HOCS complex structure within the nucleus. The basic unit of chromatin structure is the nucleosome, made up of DNA wrapped around a core of eight histone proteins (two each of H2A, H2B, H3, and H4). Another scaffolding histone protein, H1, connects these nucleosomes to form a chromatin fiber. These fiber loops create different chromatin regions or domains supported by the complex interactions among various remodeling enzymes (Me: methylation; P: phosphorylation; Ac: acetylation).

In eukaryotes, the nucleus regulates gene activation in a development- and tissue-specific manner in response to various stimuli, a phenomenon known as nuclear plasticity. Eukaryotic DNA is packaged into nucleosomes, which comprise an octamer of histone proteins and 146 base pairs (bp) of DNA [1,2,3]. The eukaryotic genome undergoes extensive and dynamic organization into hierarchically organized components, ensuring that gene expression programs are activated precisely at the appropriate time and in the appropriate tissue (Figure 1). This system design of the nucleosome, named higher-order chromatin structure (HOCS), regulates DNA replication, gene transcription, and transitions in proliferative states between actively dividing and quiescent cells [4]. The genome in eukaryotes undergoes multiple layers of regulation and comprises multiple topological levels, including chromatin loops, topologically associating domains (TADs), and A/B compartments. Any defect in the 3D genome at any layer of the organization alters the genetic and epigenetic landscape, potentially triggering diseases.

Our understanding of the physical organization of the genome in three-dimensional space has been enhanced by a wide array of technologies that map chromatin interactions. Most notably, the combination of molecular biology techniques with next-generation sequencing has enabled genome-wide epigenomic profiling by expanding our analysis capabilities from global DNA methylation levels to include the analysis of histone modifications, chromatin accessibility, interactions, and nucleosome positioning [5]. In particular, chromatin conformation capture (3C)-based techniques have revealed the three-dimensional architecture of the genome at increasing resolution, from local interactions to genome-wide contact networks [6,7,8,9]. Together with advances in genome engineering and computational modeling, such as CRISPR/Cas, these technologies have transformed our understanding of how epigenomic regulation is organized across space and scale.

**Figure 2:**
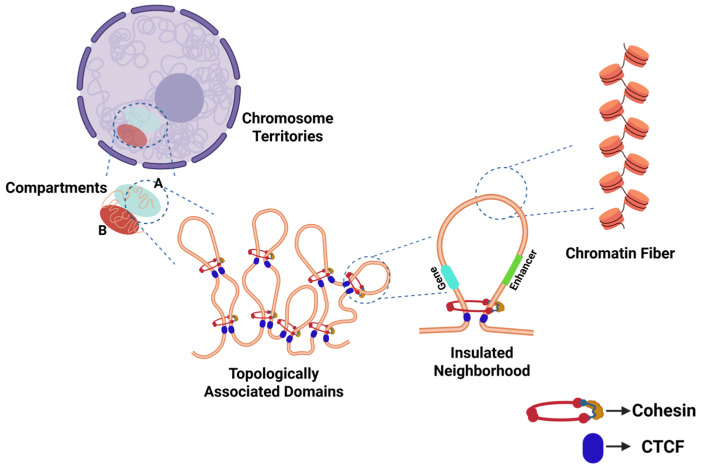
Organization of the genome in the nucleus of cells in interphase. Within distinct territories, the chromosomes are organized into transcriptionally active (**A**) and inactive (**B**) compartments. These compartments are partitioned into megabase-long topologically associated domains (TADs). Within TADs, chromatin loops are formed to allow interaction of two DNA sites, anchored by the CCCTC-binding factor (CTCF) protein and catalyzed by the cohesin complex. Such loops constitute insulated genomic neighborhoods, which facilitate precise promoter and enhancer interactions [17,18].

In the classical model of chromatin, based on data obtained in starfish spermatozoa and chicken erythrocytes, nucleosomes were proposed to fold into condensed 30 nm chromatin fibers (Figure 1) [10]. However, accumulating evidence over the past several years has challenged this view, suggesting instead that the nucleosome may be organized into clusters of dynamic, liquid-like, irregularly sized chromatin domains called topologically associating domains (TADs) without the existence of chromatin fibers. This evidence supports a chromatin structure in which TADs represent the fundamental units of higher-order chromatin structure (HOCS) in higher eukaryotic cells (Figure 2) [10,11,12,13].

The organization of chromatin structure impacts many cellular programs, such as RNA processing, and is involved in maintaining genomic integrity by facilitating DNA repair through stabilization of nuclear architecture, regulating DNA accessibility, and promoting the dynamic concentration and assembly of DNA-binding factors [14,15,16].

Beyond their role in normal genome organization, TADs have emerged as critical regulators in human disease, particularly cancer. Disruption of TAD architecture may lead to aberrant gene regulation by altering enhancer–promoter communication, thereby enabling aberrant oncogene activation. Such disruptions may arise through deletion of TAD boundaries, resulting in the fusion of neighboring domains, or through genomic rearrangements that reorganize chromatin into novel interaction domains without directly affecting boundary elements [17]. Studies in neuroblastoma, medulloblastoma, and leukemia have demonstrated that TAD disruption can function as an oncogenic driver [18,19,20,21,22]. In addition to direct structural rearrangements, mutations or dysregulation of key architectural proteins further impact higher-order chromatin organization and its vital role in maintaining genomic integrity and preventing pathological gene expression programs [23,24,25,26].

This review aims to begin with an overview of HOCS, its key regulators, and its intricate, bidirectional interplay with epigenetic mechanisms. We then aim to further discuss how HOCS influences gene expression and highlight emerging data on its implications in disease pathogenesis, particularly in cancer pathophysiology, underscoring its growing potential to redefine therapeutic approaches by targeting global chromatin regions rather than individual genes.

## 2. The Three-Dimensional Structure of the Genome

The genome’s three-dimensional (3D) structure and epigenetic regulation are deeply intertwined and mutually reinforced, as the folding of DNA into higher-order structures affects the accessibility of DNA regions for epigenetic modifications and the binding of regulatory proteins. Conversely, epigenetic modifications can alter the local structure of chromatin fibers and influence interactions between different genome regions, thereby shaping the HOCS [27]. Understanding this intricate interplay is essential for elucidating how disruption of HOCS may contribute to the development of human diseases.

The genome is hierarchically organized into functionally distinct, modular regions, which change based on epigenetic signals to allow for and to co-regulate dynamic gene expression [28]. From a functional perspective, we often categorize the chromosomal regions as falling either within active or inactive nuclear compartments (A and B compartments), with compartment A containing predominantly active euchromatin and compartment B containing largely inactive heterochromatin [28]. These compartments form the chromosome territory in the nucleus. Switching between active and inactive states, or A and B compartments, is largely regulated by the dynamically changing structure of the genome’s 3D scaffold. A crucial 3D structural process facilitating this is chromatin loop formation and dissolution. Chromatin loops are formed by interactions between distant genomic sites, bringing promoters and enhancers into close spatial proximity, contributing to gene expression, with TAD boundaries corresponding to the borders between transcriptionally active DNA and inactive heterochromatin (Figure 2) [29,30]. These loops are termed insulated neighborhoods, describing the usually megabase-sized chromatin domains separated from neighboring regions via loop formation [17]. Insulated genomic neighborhoods form the mechanistic base for TADs, which represent the fundamental structural units of higher-order chromatin organization [18]. They are crucial to many gene regulatory functions and ultimately to gene expression by bringing distant genomic regions into close spatial proximity, thus allowing distant regulatory elements to come closer to their target promoters [31]. This is especially important in instances where the enhancer is located at a distance from its target gene [32]. Dynamic long-range chromatin looping separates active and silent chromatin regions, thereby enabling the activation of the target promoter while preventing unwanted crosstalk with neighboring genomic regions [4,9,33,34,35].

**Figure 3:**
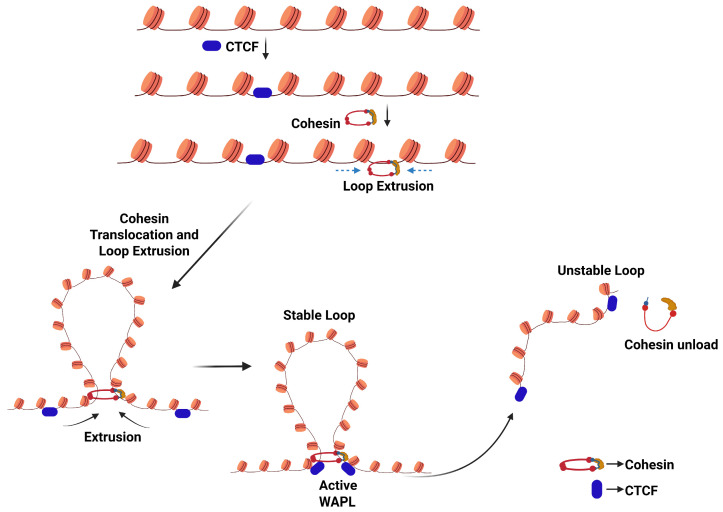
The process of establishing the CTCF/cohesin-mediated looping interactions. The chromosome is folded into loops and is anchored by CTCF. When acted upon by cohesin, these loops are progressively enlarged. The cohesin-associated protein, WAPL, regulates the cohesin-DNA interactions during mitosis and interphase. It releases cohesin from DNA and controls chromatin structure, gene regulation, and chromosome segregation.

TAD boundaries are occupied by DNA-binding protein CCTC-binding factor (CTCF) and cohesin [17]. Recent models suggest that chromatin loops in TADs are formed through loop extrusion, a dynamic and ATP-dependent mechanism where translocation of cohesin, a structural maintenance of chromosomes (SMC) protein complex, generates a progressively enlarging chromatin loop until it is stalled by CTCF, at the base of the loop, which ultimately becomes the TAD boundaries [17,36,37,38,39]. Cohesin loading and loop extrusion are facilitated by the sister chromatin cohesin 2 and 4 (SCC2/SCC4) complex (also referred to as NIPBL and MAU2), while a protein ring complex named wings apart-like protein (WAPL) promotes unloading of cohesin from the chromatin, to allow for transient loop formations (Figure 3) [37,38,39,40]. Cohesin also further stabilizes chromatin loops and plays a crucial role in chromosomal segregation and homologous recombination (HR)-associated DNA repair, as it promotes cohesion between the sister chromatids and ensures genome stability [41]. Intra-TAD looping, including regulatory enhancer–promoter loops and a few inter-TAD regulatory loops, promotes the dynamic expression of a gene (Figure 3) [42].

Beyond its role in defining TAD boundaries, CTCF is also a highly conserved transcription factor in eukaryotes and has versatile regulatory roles, such as acting as an activator, repressor, and insulator [43]. Its ability to homodimerize allows CTCF molecules to bind DNA together and serve as an anchor in forming the base of chromatin loops [33,44,45,46]. CTCF also anchors DNA to cellular structures, such as nuclear lamina, and, by creating borders at the base of the loop, limits unwanted interactions with neighboring genomic regions. This makes CTCF essential for defining TADs and maintaining gene-regulatory specificity [47]. The coordinated activity of these factors regulates the dynamic 3D architectural changes within the genome.

## 3. Key Regulators of HOCS

As described above, the CTCF and cohesin protein complexes are essential in establishing and stabilizing chromatin loops for the formation of insulated neighborhoods and TADs. Thus, both are considered master regulators of HOCS. A prominent example cited in the literature is the *Igf2/H19* imprinted locus, where CTCF-mediated binding and loop formation are critical in regulating transcription [48].

**Figure 4:**
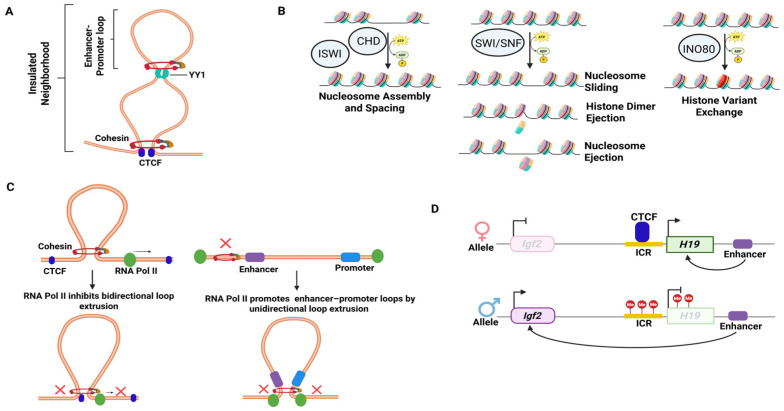
Representative regulators of higher-order chromatin structure (**A**) The transcription factor Yin Yang 1 (YY1) facilitates enhancer–promoter looping within insulated neighborhoods to control gene expression. (**B**) ATP-dependent chromatin remodeling complexes regulate genome function by modulating nucleosome positioning, composition, and occupancy. (**C**) RNA polymerase II contributes to chromatin loop establishment through two functionally distinct mechanisms: inhibition of loop extrusion (left) and promotion of enhancer–promoter loop formation (right). (**D**) Insulator activity of CTCF at the *Igf2/H19* locus on the maternal and paternal alleles. X: denotes inhibition. CHD: Chromodomain helicase DNA-binding complex. INO80: Inositol requiring 80 complex. ISWI: Imitation Switch (ISWI) complex. SWI/SNF: SWItch/Sucrose Non-Fermentable complex. ICR: Imprinting Control Region.

Additional elements involved in HOCS include the transcription factor Yin Yang 1 (YY1), which mediates dimerization in enhancer–promoter interactions (Figure 4A). Chromatin remodeling enzymes, including the Chromodomain helicase DNA-binding (CHD), Inositol requiring 80 (INO80), Imitation Switch (ISWI), and SWItch/Sucrose Non-Fermentable (SWI/SNF) complexes, also play a crucial role in regulating gene expression by modifying nucleosome positioning and DNA accessibility for CTCF binding, methylation, and demethylation [49,50]. The CHD members, CHD3, CHD4, and CHD5 constitute the nucleosome remodeling deacetylase (NuRD) complex [51,52]. The INO80 subfamily complex shifts the nucleosome exchanges with the histone dimer or variant [52]. The ISWI subfamily assembles and spaces the nucleosome, manipulating gene accessibility [50]. The SWI/SNF chromatin remodeling complexes trigger nucleosome repositioning, enabling transcription factors to access the DNA [52]. By altering DNA-histone interactions and shifting nucleosomes, these chromatin remodelers can regulate transcription by making genes accessible to transcription factors and facilitating chromatin loop interactions (Figure 4B).

Furthermore, a recent study demonstrated that RNA polymerase II (Pol II) plays a key role in forming genome-wide 3D chromatin contacts in mammals, regulating chromosomal folding and loop formation [31]. Depletion of Pol II can lead to alterations in chromatin loops, resulting in both loop gain and loss, which in turn affect enhancer–promoter interactions and ultimately alter transcription. By creating new or rewiring existing contacts with CTCF proteins, Pol II inhibits the extrusion of gained loops. A loss of loops can repress gene expression by disrupting enhancer–promoter contacts anchored by RNAPII, although Pol II depletion does not appear to affect promoter–promoter interactions or cohesin occupancy (Figure 4C) [31].

Insulators, in turn, play a pivotal role in blocking inappropriate enhancer–promoter interactions, thereby ensuring proper gene expression by maintaining the integrity of boundaries between regulatory regions. Insulators are short nucleotide sequences that serve as binding sites for sequence-specific DNA-binding proteins, forming independent domains of gene function. They act as long-range cis-regulatory elements that mediate intra- and inter-chromosomal interactions, contribute to chromosome organization by creating interactions with other insulators, and can also be involved in nucleosome modification by applying repressive histone modifications along the chromatin fiber [53,54,55,56]. In vertebrates, CTCF-related insulators, which manipulate CTCF binding through methylation of DNA sequences, are the most widely recognized and characterized insulators. An example is again the *Igf2/H19* locus, where methylation of the paternal imprinted control region (ICR) prevents CTCF from binding, but the unmethylated ICR on the maternal chromosome allows for CTCF binding, which consequently prevents the communication between the H19-proximal enhancer and the *Igf2* promoter, preventing IGF2 transcription (Figure 4D) [43,53,57].

The interplay between these chromatin-regulating mechanisms enables cells to fine-tune gene transcription in response to environmental cues, while dysregulation of these processes has been implicated not only in tumorigenesis and progression of disease but also in therapeutic resistance mechanisms across several cancers, as discussed further in Section 8. Alterations in CTCF or cohesin function and large-scale chromosomal structure rearrangements can alter gene expression programs, ultimately leading to dysregulation of transcription and activation of oncogenes without any changes to the DNA sequence [12,18,42,58,59]. Furthermore, CTCF-targeting insulators are known to inhibit enhancer activity by modulating the 3D chromatin structure, thereby influencing gene expression [57]. As a result, loss-of-function mutations in CTCF insulator genes, such as *hTERT* and *c-MYC*, alter expression patterns and contribute to tumorigenesis through oncogene activation [53]. Similarly, hypermethylation of the insulator region can block the insulator protein from binding, thereby allowing for irregular enhancer–promoter interaction, which causes abnormal gene expression [60].

Harnessing the regulatory powers of TADs and their key regulators may help define new regions of interest for reprogrammable therapeutic strategies. A major remaining limitation to realizing these advanced techniques is the pressing lack of reference 3D genomic maps for human primary normal and diseased tissues. Most of the whole-genome chromatin interaction maps have been generated using established cell lines [61]. Where primary human tissues were examined, insights into TADs and insulated genomic neighborhoods were derived from the studies of human and mouse embryonic stem cells, with only a few adult and differentiated tissues examined via high-throughput chromosome conformation capture (Hi-C) [61,62]. Notably, these studies nonetheless showed strong tissue specificity in local chromatin interactions [61]. As a result, our ability to generalize these findings to other healthy, uncharacterized tissues remains limited, and perhaps more importantly, our understanding of how these processes might be modified during aging or exploited in chronic diseases, such as cancer, remains even more constrained.

## 4. Polycomb Repressive Complexes (PRC)

Besides chromatin loops, the spatial organization of the HOCS is determined by Polycomb repressive complexes (PRCs). PRC1 and PRC2 constitute the two types of PRCs that regulate transcription in mammals, which are traditionally known as transcription repressors. PRC1 catalyzes monoubiquitylation of histone H2A at lysine 119 (H2AK119ub1), while PRC2 catalyzes the methylation of histone H3 at lysine 27 (H3K27me1/2/3), thereby marking regions for transcriptional repression and causing gene silencing [63,64].

In human and mouse embryonic stem cells, as well as human cancer-derived cell lines, PRC1-occupied genomic regions engage in long-range chromatin interactions that contribute to spatial genome organization, including at developmental gene clusters such as the HOX loci [65,66,67].

Mechanistic insights from *Drosophila* initially demonstrated that PRC1 stabilizes chromatin loops and maintains gene repression, with the loss of PRC1-associated chromatin loops leading to gene derepression [64,68]. Interestingly, newer studies in mice suggest that under certain circumstances, PRC1 can also activate transcription [69]. Moreover, EZH2, the catalytic subunit of the PRC2 complex, and RING1B, a PRC1 subdomain, have been shown to contribute to gene activation via looping effects, highlighting their dual role in both gene repression and activation and their contribution to HOCS regulation [46,70,71]. Recent research conducted in human and murine cells suggests that Polycomb proteins regulate chromatin condensation through liquid–liquid phase separation (LLPS), facilitating long-range enhancer–promoter interactions and histone modification spreading [18,72]. LLPS mediates chromatin contacts by locally concentrating PRC proteins and PRC-bound chromatin, reducing the spread of repressive histone marks [68,72]. PRC2 and PRC1 deposit H3K27me3 and H2AK119ub, respectively, and recruit the PRC1/2 variant complex onto the same chromatin regions to promote LLPS formation [72,73,74]. This is important as LLPS has been shown to weaken the insulation of a subset of TAD boundaries with high transcriptional activity in a CTCF-independent manner [75]. LLPS capabilities have been implicated in oncogenic transcription factors, such as NUP98-HOXA9 [76] and EWS-FLI [77]. Similarly, PRC1 has been implicated in embryonic development, as it has been shown that the dynamics of RING1B binding affect PRC1-regulated looping during embryonic stem cell (ESC) differentiation in mice [64,78].

In summary, PRC1 and PRC2 regulate chromatin organization in mammals through histone modifications and 3D chromatin interactions, contributing to gene repression and activation. Thus, their dysfunction can lead to developmental diseases and cancer. However, similarly to our understanding of TADs and insulated genomic neighborhoods, the majority of our knowledge of Polycomb repressive elements stems from studies conducted in Drosophila, limiting the applicability of this data to human systems, and our understanding of the role and the chromatin interactions of mammalian PRCs remains far less characterized [79]. Greater insight into these processes may lead to the development of novel therapeutics for a range of pathological conditions.

## 5. HOCS and Epigenetic Players in Aging

Chromatin undergoes significant structural and functional changes as organisms age, contributing to aging and age-related diseases. The boundaries between individual aging hallmarks are inherently blurred because they interact extensively and influence one another. López-Otín et al. proposed that aging is driven by biological processes or hallmarks that meet three criteria: (i) the time-dependent emergence of alterations accompanied by the aging process, (ii) the feasibility of accelerating aging by experimentally intensifying the hallmarks, and (iii) the ability to therapeutically target these hallmarks to slow down, halt, or reverse the process of aging. Based on these criteria, 12 key hallmarks of aging have been identified: altered cellular communication, chronic inflammation, deregulated nutrient-sensing, disabled autophagy, dysbiosis, epigenetic alterations, genomic instability, loss of proteostasis, mitochondrial dysfunction, senescence, exhaustion of stem cells, and telomere attrition [80]. Key changes include epigenetic drift [81,82], loss of heterochromatin integrity [83,84], and altered chromatin remodeling [80,85], which collectively lead to genomic instability, disrupted gene expression, and cellular senescence. For example, aging-associated chromatin changes include loss of heterochromatin protein 1 (HP1) and disrupted TAD organization, which can cause loop alterations, influencing gene regulation and cellular aging [81]. Investigating these chromatin alterations is crucial, as it offers insights into the molecular mechanisms of aging, highlights potential therapeutic targets, and paves the way for interventions that promote healthy aging. Epigenetic modifications are profoundly involved in the aging process. Several components of the complex systems responsible for generating and maintaining the epigenetic function, such as DNA methylation, histone modification, derepression of retrotransposons, non-coding RNAs (ncRNAs), and chromatin remodeling, have been linked to aging [61,80].

Research on several inherited conditions that lead to premature aging in affected individuals suggests a critical role of epigenetics, particularly chromatin remodeling, in aging. For example, Cockayne syndrome is a rare genetic disorder caused by a mutation in a chromatin remodeling protein called Cockayne syndrome group B protein (CSB). This protein is part of the SWI/SNF complex family, specifically SWI2/SNF2. The mutation of this gene results in increased reactive oxygen species (ROS) and dysregulation of the CTCF/CSB interaction under oxidative stress. CTCF acts as an architectural protein in forming chromatin loops, thereby regulating DNA topology [43]. Similarly, in Hutchinson–Gilford progeria syndrome (HGPS), a mutated nuclear membrane protein, Lamin A, results in the loss of heterochromatin protein 1 (HP1), which binds to histone H3K9me3 and is essential for heterochromatin stability [86].

These findings have led to further discoveries highlighting the importance of these epigenetic players in physiological or “chronological” aging. Disturbance in HP1 function or reduction in HP1 levels is associated with the loss of heterochromatin integrity, nuclear decondensation, and global architectural changes commonly observed in aging. Both chronologically and prematurely aged cells show reduced levels of H3K9me3 and HP1 [83]. The process of cellular senescence, one of the hallmarks of aging, has been connected to reduced levels of CTCF in animal models through loss of p16INK4a silencing due to a reduction in chromatin loop formation at its promoter site. p16INK4a drives the irreversible cell cycle arrest characteristic of senescence [87,88]. CTCF has also been implicated in tumorigenesis and cancer progression through multiple pathways, which are described in more detail in the following sections of this review [89].

Emerging research underscores the growing focus on the role of TADs in chromatin regulatory elements and aging [89]. As organisms age, dynamic changes in 3D chromatin architecture affect interactions within and between TADs [88]. Alterations in chromatin looping and TAD boundaries due to disruption of key regulators, such as CTCF and the cohesin complex, drive age-related gene expression and functional changes [90,91]. Decreased cohesin levels with aging have also been linked to female reproductive aging and defects in meiotic segregation [88]. Changes in chromatin structure directly affect the efficiency of the DNA repair machinery, contributing to genomic instability over time. The NuRD/Mi-2 complex plays a key role in DNA repair mechanisms, and aged cells have been found to lose NuRD expression, likely contributing to the development of genomic instability [92,93]. Additionally, epigenetic modifications, including DNA methylation and histone modifications, regulate chromatin structure and gene expression patterns that are associated with aging [93]. Therefore, investigating the interplay between TADs, chromatin regulatory elements, and epigenetic modifications provides valuable insights into the molecular mechanisms underlying aging and may open avenues for potential interventions to modulate the aging process and promote healthier aging.

## 6. TADs and Epigenetic Regulation of Circadian Rhythm

Interestingly, TADs are also critically involved in the regulation of circadian rhythms. Circadian rhythms regulate vital physiological functions and are synchronized by a hierarchically organized system originating in the brain. Proper synchrony between central and peripheral clocks ensures well-being and a healthy bodily state, while disrupted circadian rhythms can trigger or exacerbate metabolic diseases, psychiatric disorders, and cancer [94,95,96]. The mammalian circadian clock is based on interconnected transcriptional and post-translational feedback loops, creating oscillating cycles of transcription activation and repression. The mammalian circadian clock is built on a negative feedback loop. In this loop, the heterodimeric transcription factor circadian locomotor output cycles kaput (CLOCK) and the brain and muscle Arnt-like protein-1 (BMAL1) drive the expression of its inhibitors, the period and cryptochrome proteins [97], using histone H3 acetylation to control their expression [98].

Rev-erb family orphan nuclear hormone receptors regulate transcription in the opposite phase, executed through circadian histone acetylation and deacetylation at CRY and PER promoters, which are regulated by NAD(+)-dependent deacetylase sirtuin 1 (SIRT1) and the CLOCK protein’s intrinsic histone acetylase (HAT) activity [98]. This highlights the critical role of histone acetylation in the regulation of the circadian rhythm.

To understand enhancer–promoter interactions and genome topology in mouse liver, researchers have utilized techniques based on Hi-C and circular chromosome conformation capture (4C) sequencing strategies [99,100]. These studies reveal that liver-specific enhancer–promoter interactions are coordinated by circadian chromatin loops in a cis-fashion, initiating transcription. The molecular clock’s repressive arm rhythmically modulates chromatin loops to regulate the transcription of clock-controlled genes [99]. The core repressive transcription factor, Rev-erbα, counteracts the formation of functional loops between its regulated enhancers and circadian target gene promoters. Circadian gene expression fluctuations in the liver temporally coordinate metabolic processes such as lipid and glycogen metabolism, maintaining homeostasis [101]. A study using genome-wide and promoter-capture Hi-C to measure spatial chromatin conformation changes in mouse liver found that TADs containing circadian genes tend to switch between transcriptionally active and inactive compartments at various times throughout the day, ensuring stable boundary structures [102]. Circadian gene promoters establish maximal chromatin contacts during peak transcriptional output. Additionally, the anchor sites of circadian gene promoter loops are enriched with DNA-binding sites for liver nuclear receptors and other transcription factors [102]. Since circadian rhythm heavily relies on environmental cues, a deeper understanding of epigenetic mechanisms in animal models that simulate human conditions may pave the way for drug development targeting lifestyle disorders connected to circadian rhythm and epigenetic regulation.

## 7. TADs in Development and Congenital Diseases

Although TADs are reported to remain stable during later stages of development, there is little knowledge about how these structures correlate with gene expression. Since disruption of 3D structure in particular loci, either by genomic rearrangements or by TAD boundary alteration, triggers pathological conditions like developmental disorders or cancer, it becomes clear that maintenance of genome architecture is critical for gene expression [103].

During early development, TADs become undetectable, followed by a phase of progressive chromatin folding re-establishment. Although there are variations in the zygotic genome activation (ZGA) across species, chromatin structuring into TADs is a conserved process, underscoring its importance to developmental processes across species and time. In mammals, chromatin is undetectable before ZGA, and chromatin structure is re-established after ZGA [104,105,106].

The dynamics and stability of TADs and enhancer–promoter loops are highly context-specific. TADs are stable in both embryonic stem cells and differentiated cells [107,108,109]. However, a study showed a high variability of TAD boundaries across 37 human cell types, underscoring the importance of context-specific structural variation in TADs in development and tissue-specific gene expression. Recently, studies have established a link between structural variations and disease. For example, deletions, duplications, and inversions at the Epha4 locus disrupt TAD structure due to de novo enhancer–promoter interactions leading to misexpression of the genes *Wnt6*, *Ihh*, and *Pax3*, which results in limb malformations, like brachydactyly, polydactyly, and F-syndrome [110]. Similarly, duplications covering the neighbor *Kcnj2* gene at the Sox9 locus form a “neo-TAD” that misregulates *Kcnj2* and produces a rare limb malformation phenotype called Cooks syndrome [111]. Rearrangements within the TAD border and several retinal enhancers within the locus of the GDPD1 gene trigger an autosomal-dominant retinitis pigmentosa [112]. Finally, inversions at the TFAP2A locus disconnect this gene from its neural crest-specific enhancers and reduce its expression, causing branchio-oculo-facial syndrome [113].

In addition, irregularities involving chromatin-binding and structural proteins, caused by disruptions in TAD formation regulation, are also associated with congenital pathologies [110]. For example, mutations in SMAD2, TBX proteins, and histone acetyltransferase variants have been connected to congenital heart diseases. Disruptions in PRDM6 can lead to persistent ductus arteriosus, while irregular methylation patterns have been found in twins with Tetralogy of Fallot and double outlet right ventricle [114].

Additionally, alteration of CTCF is also implicated in multiple pathological conditions [57]. Notably, its dysregulation has been linked to several trinucleotide repeat expansion diseases, such as Huntington’s disease, fragile X intellectual disability, and myotonic dystrophy—all of which arise from excessive lengthening of microsatellite repeat sequences [115]. Mutations in a CTCF-binding site near a repeat contribute to genomic instability and the development of such diseases [116]. CTCF also plays a critical role in two other human congenital syndromes, Silver–Russell syndrome (SRS) and Beckwith–Wiedeman syndrome (BWS) [117].

Lastly, structural proteins are vitally important to the correct functioning of TADs. Human disorders arising from defects of these structural proteins are known as cohesinopathies or laminopathies, depending on the family of proteins involved. Cohesin proteins are important for mitotic chromosome condensation, sister chromatid cohesion, gene regulation, DNA repair, 3D genome organization, cell cycle, apoptosis, and tissue development. Mutated cohesin subunits and their regulators lead to a spectrum of developmental and intellectual impairment disorders. Mutations in cohesin components result in three major cohesinopathies—Cornelia de Lange syndrome (CdLS) [118,119,120], Roberts syndrome (RBS), and Warsaw breakage syndrome (WABS) [121,122,123]—which are characterized by limb defects, craniofacial deformities, growth retardation, intellectual disability, cardiac malformations, and microcephaly.

The nuclear lamina provides structural support to the nucleus and is essential for maintaining nuclear structure and 3D genome organization. The lamina comprises three types of filamentous protein, (lamin A, B, and C, with multifarious roles in several nuclear processes and cellular pathways. Mutations in the intermediate filament nuclear lamins and lamin B receptor (LBR) genes constitute rare laminopathies [86]. Although cohesinopathies and laminopathies are multisystem monogenic diseases, laminopathies exhibit different mutations in the same gene, producing different disorders. A mutated *LMNA* encoding the A/C-type lamins causes a large spectrum of disorders from premature aging syndromes (Hutchinson–Gilford progeria syndrome (HGPS) [86] and Werner syndrome) to myopathies (autosomal forms of Emery–Dreifuss muscular dystrophy (EDMD), limb-girdle muscular dystrophy type 1B (LGMD1B), and dilated cardiomyopathy type 1A (DCM1A)), neuropathies (e.g., Charcot–Marie–Tooth type 2B1 (CMT2B1)), and lipodystrophies (e.g., Dunnigan-type familial partial lipodystrophy (FPLD)).

In addition to the mentioned proteins, there are other architectural proteins with critical implications in various diseases, as listed below (Table 1).

Deeper knowledge of epigenetic involvement in congenital disorders may enhance and broaden treatment options and targeted gene therapies for patients with rare congenital syndromes.

## 8. TADs and Chromatin Regulatory Elements in Cancer

In human cancers, epigenetic enzymes, like SET and SETBD1, are often overexpressed and contribute to chromatin compaction [124,125]. In isocitrate dehydrogenase 1/2 (*IDH1/2*)-mutated human malignancies, suppression of histone demethylation by accumulated 2-hydroxyglutarate results in hyper-trimethylation of histone H3K9, which makes them exquisitely sensitive to the PARP inhibitors [126]. A recent study has shown that an RIF1-ASF1 histone chaperone complex formed in response to DNA damage promotes HOCS changes, stimulating the NHEJ pathway for DSB repair and conferring the BRCA1-deficient cells with sensitivity to PARP inhibitors [127]. Anthracycline drugs have been reported to function as epigenetic modulators that target the 3D genome architecture in chronic myeloid leukemia [128]. A new class of epigenetics-based anti-cancer drugs called bromodomain and extraterminal protein (BET) inhibitors, which block oncogene transcription by preventing BETs from reading acetylation marks on histones, are actively being investigated in pre-clinical and clinical trials [129,130,131,132]. This highlights the promising prospect that epigenetic enzymes can serve as biomarkers and as targets in cancer therapy.

More recently, TAD disruptions have become an additional focus of investigation as they have been found to play a role in cancer pathogenesis and progression as well, with several mechanisms proposed. Oncogene activation can be triggered by mutations or epigenetic modifications affecting the gene or its regulatory elements. TADs are often disrupted in cancers and “drive” oncogenesis by either of the two mechanisms: 1. Through local disruption of domains via TAD boundary deletion, which fuses the two adjacent TADs; or 2. By breaking up TADs through genomic rearrangements to create new domains without directly affecting the TAD boundary [133]. TAD disruption can act as an oncogenic “driver” mutation, as evident in neuroblastoma [19,20], medulloblastoma [21], and leukemia [18,22].

Besides altering the arrangement of genes and boundaries within genomic TADs, the emergence of multiple pathological phenotypes in humans can be triggered by mutations in the coding sequence or misexpression of the TAD-associated architectural proteins cohesin and CTCF [134]. Somatic mutations in cohesin or CTCF, both considered tumor suppressor genes, have been associated with various types of cancer [23,24,25,26]. Therefore, regulating genes and preventing pathological conditions rely heavily on the crucial involvement of both cohesin and CTCF.

As previously discussed, cohesins are vital in establishing and maintaining the pairing of sister chromatids during DNA replication and mitosis [135,136]. Mutated cohesin weakens its association with genomic target sites and interacts with CTCF to interfere with its boundary and insulator function [135]. Depletion of cohesin in human cells alters the expression of several hundred genes, which therefore makes it an attractive potential target of therapeutic intervention [137,138]. Among the cohesin subunits, STAG2 has been identified as a therapeutic target in cohesin-mutated cancers [138], and given its high mutation frequency, likely particularly in myeloproliferative malignancies, as it was shown to be essential for DNA replication fork progression, with STAG2 inactivation leading to collapse of the replication machinery [41]. Additionally, mutated STAG2 has been implicated in Ewing sarcoma (EWS), a pediatric tumor of the bone and soft tissues [41] and studies in advanced bladder cancer revealed drug sensitization to PI3K inhibitors in bladder cancers with STAG2 loss-of-function mutations [139]. Another cohesin subunit, RAD21, has been identified as an oncogene facilitating immune evasion through suppression of interferon signaling in ovarian cancer. The same study also found that in the case of successful RAD21 activation, the sensitivity of ovarian cancer cells to PARP inhibitors increased significantly, marking RAD21 as a therapeutic target with high potential [140]. Increased expression of RAD21 in mesenchymal cancer and lung cancer cells has also been described to promote the epithelial-to-mesenchymal transition (EMT), making it a therapeutic target in these cancers, and studies in lung adenocarcinoma have shown that treatment with ERK inhibitors was able to reverse RAD21-driven metastasis [141,142,143].

Heterochromatin protein 1 (HP1), a structural component of heterochromatin, has been implicated in cancer, given its role in transcriptional repression [144,145]. Levels of HP1 isoforms are altered in various types of cancers, including breast, brain, colon, and ovary, and their expression levels appear to play a significant role in cancer progression [146]. For example, HP1Hsalpha is reported to be downregulated in highly invasive and metastatic breast cancer cells compared to less invasive and nonmetastatic counterparts [147,148]. Another isoform of the HP1 protein, HP1γ (Cbx3), is a key epigenetic regulator that is markedly upregulated in several cancers, including breast, colon, lung, pancreas, gastric, castration-resistant prostate, and renal cancers, where it is implicated in driving cancer progression [149,150,151,152,153,154,155].

SATB1 (Special AT-rich binding protein 1) is another DNA-binding protein that exists explicitly in thymocytes and binds to AT-rich sequences, and in response to superhelical tension, this protein unwinds DNA strands [156]. Its expression in breast cancer is correlated with poor prognosis, as it promotes aggressive growth of breast cells in vitro and regulates numerous genes involved in cell adhesion, cellular signaling, and cell-cycle regulation [157]. In head and neck squamous cell carcinoma, it was identified as an oncogenic driver, and in vivo trials with polymeric nanoparticles containing siRNA directed against SATB1 showed successful inhibition of tumor growth in human xenograft-bearing mouse models [158]. Meanwhile, evidence suggests that SATB1 may have a tumor suppressor function in human cutaneous T cell lymphoma (Sézary syndrome), and treatment with certain methyltransferase inhibitors was shown to selectively stunt the growth of human primary Sézary cells (isolated from patient donor samples) in vitro [159].

Genomic rearrangements through disruption of chromatin architecture can lead to gene fusion and subsequent overexpression and function as oncogenes. In alveolar rhabdomyosarcoma, for example, the genes *PAX3* and *FOXO1* fuse when both regulatory loops (RLs) fuse and activate *PAX3* transcription via enhancers from the *FOXO1* RL [160]. Other examples are listed in Table 2. HOCS can modulate interactions between different loci spanning several megabases, forming “megadomains” in a highly aggressive type of squamous cell carcinoma. These megadomains undergo genomic translocation due to the fusion of BRD4-NUT oncoprotein and form large hyperacetylated chromatin by recruiting p300. Upregulation of intra- and interdomain interactions and SOX2, TP63, and MYC expression triggers tumorigenesis [161,162,163].

Besides genomic rearrangements, TAD boundary alteration can also lead to tumorigenesis in a gene expression-independent manner. The Cancer Genome Atlas has revealed that only 14% of cancer-associated TAD boundary deletions significantly alter the expression of nearby genes [59]. In gliomas, TAD boundary disruption is observed due to gain-of-function mutations in the *IDH* gene. These disruptions activate key cancer drivers and lead to long-range interactions between genes and enhancers outside the TAD. Mutations near oncogenes have been reported in ovarian and colorectal cancers, often leading to the disruption of TADs, resulting in subsequent miscommunication between genes and their regulatory elements. In T-ALL, for example, deletions near oncogenes delete TAD boundaries, activating proto-oncogenes *TAL1* and *LMO2* [18,133,164]. Globally, rearrangements of TAD boundaries can cause genomic rearrangements, triggering cancer formation. Further studies have outlined inversion events that disrupt two TADs at breakpoints and cause inappropriate activation of oncogenes such as *EVI1* [22,165].

CTCF-binding site mutations often cause nearby genes to misexpress, leading to tumorigenesis [166]. In melanoma, overexpressing neurotensin leads to a gain in the CTCF-mediated chromatin loop, enhancing the contact between the *NTS* promoter and a CRE in the intron of *LRRIQ1*, demonstrating that both loss and gain of chromatin structures can have detrimental consequences [167]. CTCF binding also influences the expression of other cancer-relevant genes, including tumor suppressors *RASSF1A* and *CDH1* [168]. *CDH1* mutation status has been shown to influence sensitivity to treatment with PARP inhibitors in gastric cancer [169]. These findings demonstrate the importance of CTCF in preventing tumorigenesis by establishing and maintaining the higher-order chromatin structure (HOCS) around tumor suppressor genes and its vast implications as a potential therapeutic target. Moreover, alterations in DNA methylation within CTCF-binding sites disrupt CTCF-mediated loop formation, leading to aberrant enhancer–promoter interactions in glioma, gastrointestinal stromal tumors, and T-ALL [52].

In a recent study, it was reported that an increase in CTCF site methylation in mutant *IDH* cells partially inactivated TAD boundaries, simultaneously activating key cancer drivers such as PDGFRA by enhancers located outside the normal PDGFRA TAD [133]. TAD boundary inactivation was found to depend on DNA methylation, resulting in long-range interactions between PDGFRA and a constitutive enhancer outside the TAD. In another study, single-nucleotide polymorphism-associated diseases were often found at or near gene regulatory elements but rarely at TAD boundaries. In contrast, cancer cells exhibited severe alterations in TAD boundaries and their CTCF DNA-binding motifs [133]. Another study discovered that CTCF sites adjacent to oncogenes are frequently mutated, as seen in ovarian cancer, where a mutated CTCF motif at the boundary of the TAD containing the *NOTCH1* gene causes *NOTCH1* misregulation due to inappropriate enhancer action caused by a disrupted TAD [170]. Comprehensive mutation analysis in colorectal cancers revealed that CTCF-binding sites binding cohesin are the most frequently mutated, often leading to disruption of TADs and breach of communication between genes and their distal regulatory elements. As mentioned above, in T-ALL, TAD boundary deletions near the *TAL1* and *LMO2* proto-oncogenes have been found to cause their oncogene activation [164]. These findings underscore the importance of TAD boundary disruptions in tumorigenesis but also highlight them as a therapeutic target. Recently, an epigenetic-based anti-cancer drug called Curaxin CBL0137 was developed to specifically target the 3D chromatin organization by disrupting long-range cis-regulatory element interactions and inducing partial deletion or loss of CTCF from genomic binding sites [171].

Ultimately, further molecular insights into the unique patterns of TAD in cancer might enhance our understanding of the genetic basis of gene expression and provide specific targets for treating many of the above-discussed cancers in the future.

**Table 2:** This table shows various syndromes caused by chromosomal rearrangements of certain genes responsible for maintaining the architecture of the chromatin.

Syndrome/ Disease	Gene Involved	Salient Features	References
Type A1 brachydactyly	*PAX3*	Deletion	[172]
Cook’s syndrome	*KCNJ2*	Duplication	[111]
Rett syndrome	*FOXG1*	Translocation	[173]
Glass syndrome	*SATB2*	Translocation	[173]
5q14.3 microdeletion syndrome	*MEF2C*	Deletion	[173]
Autosomal-dominant adult-onset demyelinating leukodystrophy (ADLD)	*LMNB1*	Deletion	[35]
Mesomelic dysplasia	*ID4*	Deletion	[172]
Liebenberg syndrome	*PITX1*	Deletion, duplication	[172]
Glioma	*PDGFRA*	Hypermethylation of the CTCF-binding site	[174]
Colorectal cancer	-	Mutations in the CTCF/cohesion-binding site	[166]
T cell acute lymphoblastic leukemia (T-ALL)	*TAL1, LMO2*	Deletion of CTCF/cohesion-binding sites	[164]
Neuroblastoma	*TERT*	Diverse SVs	[19]
Lung cancer	*IRS4*	Deletion	[175]
Colorectal cancer	*IGF2*	Tandem duplications	[175]
Medulloblastoma	*GFI1, GFI1B*	Diverse SVs	[21]
AML with inv(3)/t(3;3)	*EVI1*	Inversion	[22]
Head and neck squamous cell carcinoma	*SATB1*	Oncogene	[158]
Myeloproliferative neoplasms and bladder cancer	*STAG2*	Depletion of cohesin	[138,139]

## 9. Assessing Chromatin Regulatory Elements: Technical Advances and Limitations

This last decade has witnessed remarkable advancements in molecular and computational methods for analyzing HOCS regulatory elements. Methods like chromatin immunoprecipitation, accessibility, and DNA methylation studies have enabled annotation of regulatory elements and explored the interactions between transcription factors and regulatory elements. The evolution of chromosome conformation capture–sequencing-based approaches allowed for deeper mapping of chromatin, and after the development of Hi-C, which enabled genome-wide chromatin interaction mapping, expanded our understanding of chromatin interactions even further.

However, as our understanding of the 3D genome and its regulatory roles in the various nuclear processes remains in early stages, further studies and new techniques are necessary. New mapping technologies like the 4D Nucleome (4DN) project focus on the spatial resolution of the 3D genome [176]. It aims to study the genome’s spatial organization and determine its effect on gene regulation through experimental and computational approaches. Moreover, genome editing studies would help determine the functional consequences of perturbing structural features in various biological contexts. Such experiments would help provide deeper mechanistic insights into the 3D genome organization, supporting how perturbation of the different organizational features would affect nuclear activities, and understanding the molecular mechanisms of disease upon the 3D genome disruption [165].

A major challenge in this area, in our opinion, is the construction of high-resolution reference maps of the 3D genome in human disease cells and their corresponding normal tissue cells, which are necessary for studying chromatin interaction. Population-based Hi-C studies are constrained by the cellular and genetic heterogeneity of primary disease samples, and it therefore appears equally important to statistically survey large populations of single cells to study cell-to-cell variability of chromosome conformation. However, currently available single-cell Hi-C methods remain limited in throughput or resolution. Overall, continued development of high-throughput and high-resolution single-cell Hi-C methods will be crucial in dissecting the aberrant 3D genome architecture across heterogeneous human tissues. Various ATP-dependent chromatin remodeling complexes are involved in long- and short-distance chromatin interactions by regulating the appropriate nucleosome organization for CTCF and cohesin complexes. Although remodelers often recruit cohesin-loading machinery, their roles in mammals are still unclear. Further studies on the role of remodelers on the 3D structure require using acute depletion/inhibition of specific remodelers or CRISPR-mediated contact sequence modifications combined with high-resolution techniques, such as ChIA-PET or Hi-ChIP, will be essential for clarifying their contributions to genome architecture.

Species ranging from bacteria to mammals harbor abundant repetitive DNA sequences covering almost half of the genome. It is important to note that in NGS, these repetitive elements have always posed significant technical challenges for sequence alignment and assembly. Short read lengths and massive data volumes generated by NGS libraries further exacerbate these difficulties. Hence, from a computational standpoint, these repetitive sequences create ambiguities in read alignment and assembly, leading to biases and potential errors during downstream data analysis. However, from a biological perspective, these often-ignored repeats encode critical regulatory and functional information and therefore cannot be overlooked [177]. Notably, more than 25 inherited human disorders are caused by the unstable expansion of repetitive DNA sequences known as short tandem repeats (STRs) [178]. However, 3D genome mapping-based assays, such as Hi-C assays, are unable to adequately account for reads that align to multiple genomic locations. As such, biological signals from repetitive sequences critical for tissue specificity, and the onset of human diseases and disorders, remain systematically underrepresented [179].

With continued technological advancements, integrating Hi-C with other next-generation sequencing methods will allow for detailed analysis of the genome-wide chromatin structure, promoter–enhancer interactions, and structural rearrangements.

## 10. Conclusions and Prospects

In this review, we have highlighted the critical roles of genome organizers in human development, tissue regeneration, aging, and cancer pathophysiology.

The study of HOCS has become a focus of investigation, which is particularly interesting for disease detection, monitoring, and surveillance. Unlike approaches that focus solely on individual genes or proteins, examining higher-order chromatin has the potential to uncover global HOCS-based signatures that can distinguish between healthy and diseased cells or identify cell states within heterogeneous tissue sources. The unprecedented understanding of genomic spatial organization underscores its translational and clinical significance. We discussed key exemplary studies among the growing body of evidence supporting HOCS machinery as promising targets for cancer therapy [180].

Despite the growing abundance of epigenomic datasets, further research is necessary to map the regulatory elements and chromatin structures across diverse tissues and disease states to identify novel 3D epigenetic signatures. Given the dynamic epigenetic profiles and cell-type-specific nature of HOCS regulatory pathways—shaped by time, environmental exposures, and disease states—understanding these mechanisms may represent one of the “master keys” to advancing precision medicine.

In summary, exploring higher-order chromatin structure presents a promising avenue for transforming cancer diagnosis and therapeutic development. By viewing chromatin structures as “zip codes”, where single genes represent “houses”, researchers can gain insights into the global genomic landscape associated with specific diseases. This approach moves beyond the traditional gene-by-gene analyses, offering a more comprehensive understanding of the complex regulatory networks and highlighting the potential of targeting chromatin architecture to enable more effective and personalized treatments in the shift toward precision medicine and precision oncology.

## Figures and Tables

**Table 1:** List of diseases/syndromes associated with the defects in the various chromatin architectural proteins and their inheritance mode.

Chromatin Architectural Protein	Syndrome/Disease	Mode of Inheritance	OMIM ID
CTCF	Autosomal dominant intellectual disability 21 (MRD21)	Autosomal dominant	615502
ESCO2	Roberts syndrome	Autosomal recessive	268300
Lamin A	Hutchinson–Gilford progeria syndrome (HGPS)	Autosomal dominant	176670
RECQL2 or WRN	Werner syndrome (WS)	Autosomal recessive	277700
Lamin A/C	Emery–Dreifuss muscular dystrophy 2 (EDMD2)	Autosomal dominant	181350
Lamin A/C	Emery–Dreifuss muscular dystrophy 3 (EDMD3)	Autosomal recessive	616516
Lamin A/C	Limb-girdle muscular dystrophy type 1B (LGMD1B)	Autosomal dominant	159001
Lamin A/C	Dilated cardiomyopathy type 1A (DCM1A)	Autosomal dominant	115200
Lamin A/C	Charcot-Marie-Tooth type 2B1 (CMT2B1)	Autosomal recessive	605588
Lamin A/C	Dunnigan-type familial partial lipodystrophy (FPLD)	Autosomal dominant	151660
MED12	FG syndrome	X-linked recessive	305450
MED12	Fryns-Lujan syndrome	X-linked recessive	309520
NIPBL	CdLS1	Autosomal dominant	122470
SMC1A	CdLS2	X-linked dominant	300590
SMC3	CdLS3	Autosomal dominant	610759
RAD21	CdLS4	Autosomal dominant	606462
LBR	Pelger–Huët anomaly (PHA)	Autosomal dominant	169400
LBR	Greenberg dysplasia	Autosomal recessive	215140

## Data Availability

No new data were created or analyzed in this study.
